# Spatiotemporal Dynamics of *Vibrio* spp. within the Sydney Harbour Estuary

**DOI:** 10.3389/fmicb.2016.00460

**Published:** 2016-04-12

**Authors:** Nachshon Siboni, Varunan Balaraju, Richard Carney, Maurizio Labbate, Justin R. Seymour

**Affiliations:** ^1^Plant Functional Biology and Climate Change Cluster, University of Technology Sydney, UltimoNSW, Australia; ^2^School of Life Sciences, The ithree institute, University of Technology Sydney, UltimoNSW, Australia

**Keywords:** *Vibrio*, seasonal variation, abundance and diversity, *Vibrio cholerae*, *Vibrio vulnificus*, *Vibrio parahaemolyticus*

## Abstract

*Vibrio* are a genus of marine bacteria that have substantial environmental and human health importance, and there is evidence that their impact may be increasing as a consequence of changing environmental conditions. We investigated the abundance and composition of the *Vibrio* community within the Sydney Harbour estuary, one of the most densely populated coastal areas in Australia, and a region currently experiencing rapidly changing environmental conditions. Using quantitative PCR (qPCR) and *Vibrio*-specific 16S rRNA amplicon sequencing approaches we observed significant spatial and seasonal variation in the abundance and composition of the *Vibrio* community. Total *Vibrio* spp. abundance, derived from qPCR analysis, was higher during the late summer than winter and within locations with mid-range salinity (5–26 ppt). In addition we targeted three clinically important pathogens: *Vibrio cholerae, V. Vulnificus*, and *V. parahaemolyticus*. While toxigenic strains of *V. cholerae* were not detected in any samples, non-toxigenic strains were detected in 71% of samples, spanning a salinity range of 0–37 ppt and were observed during both late summer and winter. In contrast, pathogenic *V. vulnificus* was only detected in 14% of samples, with its occurrence restricted to the late summer and a salinity range of 5–26 ppt. *V. parahaemolyticus* was not observed at any site or time point. A *Vibrio*-specific 16S rRNA amplicon sequencing approach revealed clear shifts in *Vibrio* community composition across sites and between seasons, with several *Vibrio* operational taxonomic units (OTUs) displaying marked spatial patterns and seasonal trends. Shifts in the composition of the *Vibrio* community between seasons were primarily driven by changes in temperature, salinity and NO_2_, while a range of factors including pH, salinity, dissolved oxygen (DO) and NO_x_ (Nitrogen Oxides) explained the observed spatial variation. Our evidence for the presence of a spatiotemporally dynamic *Vibrio* community within Sydney Harbour is notable given the high levels of human use of this waterway, and the significant increases in seawater temperature predicted for this region.

## Introduction

Marine microbes play essential ecological and biogeochemical roles in coastal and estuarine habitats (Ducklow and Carlson, 1992), but some species also pose a significant threat to ecosystem function and human health. A key group of marine pathogens responsible for diseases in a wide diversity of marine organisms, as well as illness in the human population, belong to the bacterial genus *Vibrio*. The *Vibrio* genus is comprised of a diverse group of gram-negative, largely marine and estuarine heterotrophic bacteria that frequently occur in close association with marine plants and animals, where they can act as both mutualistic symbionts (Lee and Ruby, 1994; Nyholm and Nishiguchi, 2008) or pathogens (Blackwell and Oliver, 2008; Vezzulli et al., 2012, 2015; Geng et al., 2014; Matteucci et al., 2015). As an important natural component of bacterioplankton communities they also contribute to biogeochemical cycling in aquatic habitats (Eiler et al., 2006; Thompson and Polz, 2006; Hasan et al., 2015). Due to their potentially significant impact on coastal ecosystems, marine animals, aquaculture (Higgins, 2000; Kushmaro et al., 2001; Ben-Haim and Rosenberg, 2002; Zorrilla et al., 2003; Austin and Zhang, 2006; Geng et al., 2014; Vezzulli et al., 2015) and human health (Daniels and Shafaie, 2000; Baker-Austin et al., 2013; Orata et al., 2014) an understanding of the spatiotemporal dynamics of *Vibrios* and their potential to bloom and cause disease outbreaks has become increasingly important (Lipp et al., 2002; Oberbeckmann et al., 2012; Takemura et al., 2014). This is particularly true in light of recent evidence that this group of organisms are increasing in abundance and impact in some regions as a consequence of environmental perturbations and climate change (Oberbeckmann et al., 2012; Froelich et al., 2013; Jacobs et al., 2014; Takemura et al., 2014).

*Vibrios* are copiotrophic bacteria that have the capacity to rapidly increase in abundance, shifting from a relatively rare component of coastal microbial communities to dominant members of the assemblage during “bloom” events (Thompson and Polz, 2006; Gilbert et al., 2011; Takemura et al., 2014). For example, during a 1 month period, the abundance of a single *Vibrio* sp. operational taxonomic unit (OTU), increased from <2 to 54% of the bacterial community inhabiting a coastal habitat, in apparent response to an increased abundance of a single diatom species (Gilbert et al., 2011).

As well as causing diseases in fish, corals, oysters, crustaceans, echinoderms, and other animals (Goarant et al., 2000; Kushmaro et al., 2001; Ben-Haim and Rosenberg, 2002; Becker et al., 2004; Frans et al., 2011; Geng et al., 2014; Vezzulli et al., 2015) multiple *Vibrio* spp. are also pathogenic to humans (Adebayo-Tayo et al., 2011; Baker-Austin et al., 2013; Geng et al., 2014; Orata et al., 2014; Kubota, 2015). Arguably the most clinically important pathogens for public surveillance matters are *Vibrio cholerae, V. parahaemolyticus*, and *V. vulnificus* (Daniels and Shafaie, 2000). In the USA, these three *Vibrio* spp. are responsible for an economic burden of $307 million USD per year (Ralston et al., 2011). Toxigenic *V. cholerae* is the causative agent of cholera, a disease for which around 2.8 million cases occur annually around the world, with an average of ∼93,500 deaths each year (Ali et al., 2012). Infections by *V. parahaemolyticus* and *V. vulnificus* are typically associated with either the consumption of undercooked seafood, or direct contact from swimming in coastal and estuarine waters (Daniels and Shafaie, 2000). In Japan, it is estimated that between 7,000 and 63,000 people experience foodborne illnesses caused by *V. parahaemolyticus* each year (Kubota, 2015). While in the USA, the estimated economic burden of medical costs associated with *V. vulnificus* infections represents approximately half of the total costs associated with food and water-borne marine pathogens and toxins (Ralston et al., 2011). Moreover, the capacity of *V. vulnificus* to cause wound infections that become septic is particularly notable, because these infections result in a ∼50% mortality rate (Daniels and Shafaie, 2000; Jones and Oliver, 2009; Horseman and Surani, 2011).

Understanding when and where outbreaks of pathogenic *Vibrios* will occur is essential for public health management (Hlady and Klontz, 1996; Ralston et al., 2011). The spatiotemporal dynamics of *Vibrio* populations and the occurrence of bloom events have been linked to several environmental drivers, including temperature, salinity, turbidity, dissolved oxygen (DO), pH, chlorophyll, and nutrients, as well as associations with potential host organisms (Takemura et al., 2014). In coastal environments, as well as estuaries and coastal rivers, elevated water temperatures and low salinity levels are also often significant explanatory factors for increases in *Vibrio* spp. abundance (Randa et al., 2004; Hsieh et al., 2008; Oberbeckmann et al., 2012; Froelich et al., 2013; Takemura et al., 2014), though there is evidence that the key environmental drivers can differ between species (Takemura et al., 2014). *V. cholerae, V. vulnificus*, and *V. parahaemolyticus* all generally prefer warm water temperatures (>20°C) and low (<10 ppt) salinity (Takemura et al., 2014), with infectious *Vibrio* outbreaks often tightly correlated with these parameters (Oberbeckmann et al., 2012; Jacobs et al., 2014; Takemura et al., 2014).

As a result of increasing sea surface temperatures driven by climate change, the Baltic Sea in Northern Europe has seen an increase in *Vibrio*-related wound infections with spikes in disease correlating with “heatwave” years (Baker-Austin et al., 2013). Similar correlations between *Vibrio* abundance (and infections) and temperature increases have been made in other parts of the world including Israel, the USA, Chile, Peru, and Spain (Centers for Disease Control and Prevention, 1998; González-Escalona et al., 2005; Paz et al., 2007; Martinez-Urtaza et al., 2008; Baker-Austin et al., 2010). It has been reported that an increase in sea surface water temperature of 3.7°C increases *Vibrio*-associated illness risk by 2–3 times (Sterk et al., 2015), while a 5°C temperature rise increases cholera risk by 3.3-fold (Huq et al., 2005; Colwell, 2009; Baker-Austin et al., 2013).

The links between pathogenic *Vibrio* spp. abundance and elevated seawater temperatures are particularly pertinent to south-eastern Australia, where seawater temperatures are currently rising more rapidly than in any other part of the southern hemisphere (Hobday and Lough, 2011). These increasing seawater temperatures are believed to be driven in part by the influence of the strengthening East Australian Current (EAC), a western boundary current that redistributes warm tropical waters into the temperate latitudes of the Australian east coast (Cai et al., 2005). The EAC is currently increasing in southerly extent as a result of climate change driven shifts in Pacific Ocean circulation patterns (Cai et al., 2005; Sun et al., 2012), and as a consequence southerly range expansions of tropical animal and plant species into the temperate latitudes of the Australian east coast have been observed (Poloczanska et al., 2007; Last et al., 2011). The EAC has also been implicated in the southward spread of populations of “tropical” microbial species into temperate latitudes of the Tasman Sea (Seymour et al., 2012).

It is predicted that by 2050 the average sea surface temperatures in south-eastern Australia will be 2°C higher than the 1990–2000 average, which will also result in higher temperature extremes during “heatwave” years (Hobday and Lough, 2011). Within the context of temperature-associated increases in the abundance of pathogenic *Vibrios* observed elsewhere (Huq et al., 2005; Oberbeckmann et al., 2012; Vezzulli et al., 2012; Baker-Austin et al., 2013; Jacobs et al., 2014; Takemura et al., 2014), current and predicted future increases in seawater temperatures raise the prospect for increased incidence of pathogenic *Vibrios* within Australian coastal waters. This is significant given that approximately 85% of the human population in this region reside within 50 km of the coast (Australian Bureau of Statistics, 2004). However, there is currently a severe lack of data regarding the natural occurrence and distributions of pathogenic *Vibrios* within the Australian marine environment or an understanding of the environmental processes underpinning them.

The Sydney Harbour estuary is one of the most densely populated coastal areas in Australia and the waters of this region are heavily used by commercial and recreational activities. This system has also been subject to significant contamination from heavy metals, organics, and a range of other pollutants (Birch, 1996; Thompson et al., 2011; Lee and Birch, 2014). The estuary hosts a diversity of habitats and high levels of biodiversity (Hutchings et al., 2013), but little is known about the microbiology of this environment. In this study, we investigated the dynamics of *Vibrio* spp., including human pathogen species, within Sydney Harbour.

## Materials and Methods

### Study Sites

This study was conducted within the Sydney Harbour estuary system, located on the central eastern coast of Australia (33°48′S 151°18′E). The Sydney Harbour estuary system spans an area of approximately 250 km^2^, and is completely encircled by the city of Sydney in all directions except to the east, where it meets the Tasman Sea. The estuary is exposed to high levels of anthropogenic impact (Lee and Birch, 2014) and is located in the temperate region of the east Australian coast – a region experiencing rapid increases in sea surface water temperatures (Hobday and Lough, 2011).

**FIGURE 1:**
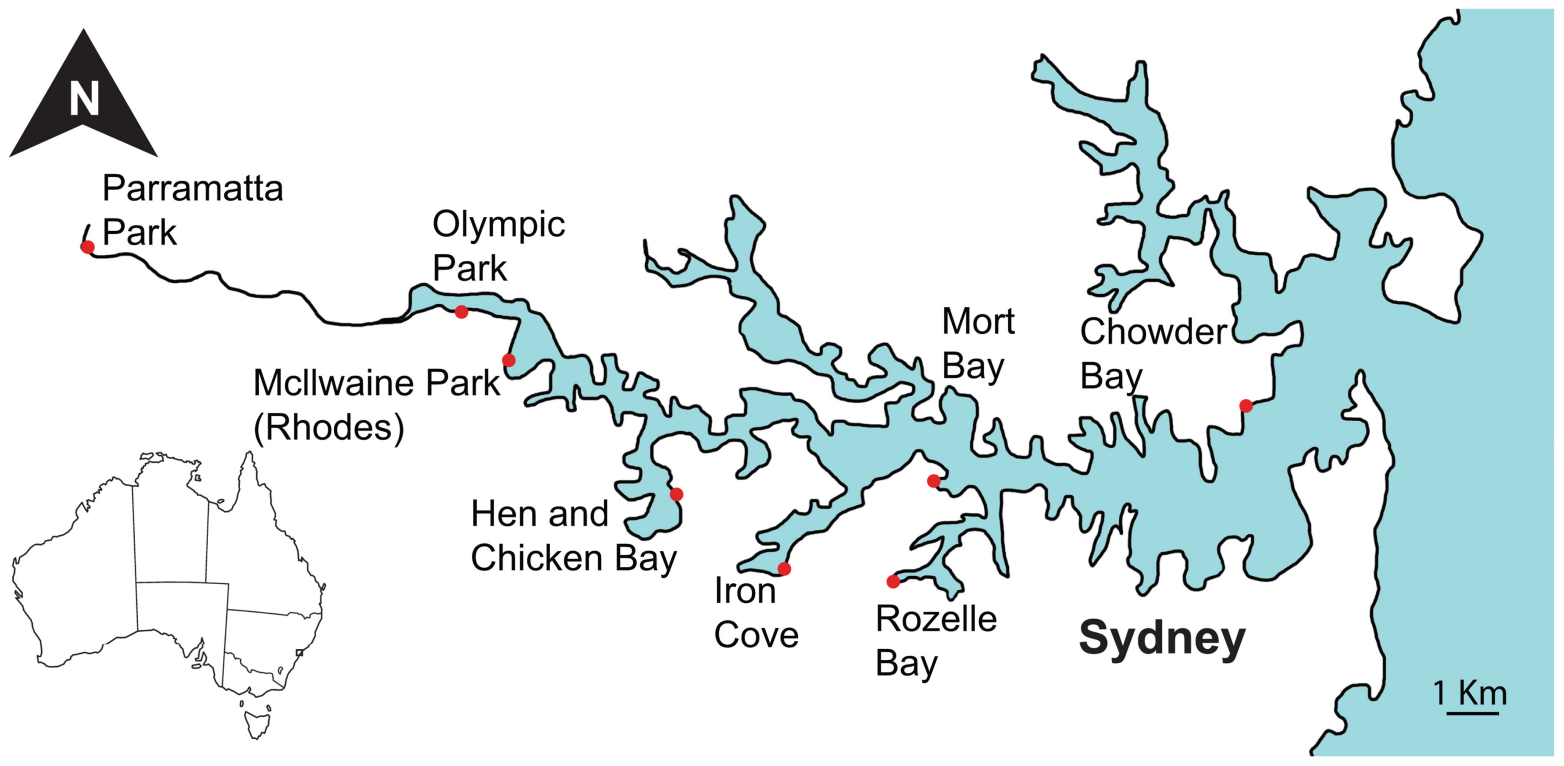
**Map of sampling locations along the Parramatta River and within Sydney Harbour**.

Sampling was performed at eight sites within the Sydney Harbour catchment and included four inner harbour and four outer harbour sites, spanning a salinity gradient from marine conditions near the mouth of the harbour at Chowder Bay (33°50′22″S 151°15′17″E) to the freshwater conditions in the Parramatta River to the west (33°48′36″S 150°59′46″E, **Figure 1**). Sites at Olympic Park (33°49′20″S 151°04′44″E) and Mcllwaine Park (Rhodes) (33°49′52″S 151°05′22″E) are situated furthest inland and near mangrove areas. The Hen and Chicken Bay (33°51′22″S 151°07′38″E) and Iron Cove (33°52′11″S 151°09′06″E) sites are directly adjacent to land areas that have been reclaimed using commercial and industrial waste (Suh et al., 2004). The sites at Rozelle Bay (33°52′20.0″S 151°10′32.6″E) and Iron Cove are adjacent to stormwater canals that are subject to regular discharges (Armitage and Rooseboom, 2000). The two most eastern sites at Mort Bay (33°51′13″S 151°11′05″E) and Chowder Bay are closest to the mouth of the Harbour. Sampling was conducted from jetties (piers) or pontoons at each site, whereby surface water samples were collected from points where the water depth was 2–5 m, with the exception of Hen and Chicken Bay where the water depth was only 1 m. Sampling was conducted on three occasions, corresponding to the late austral summer in March 2014, and the austral winter in June and August 2014.

### Sampling Protocols

At each of the sampling sites, approximately 16 L of water was collected from the surface using Nalgene bottles, and physicochemical parameters including temperature, DO, conductivity and pH were measured *in situ* using a WTW multiprobe meter (Multi 3430, Germany). Triplicate surface water samples (15 ml) were also collected at each site and filtered through 0.45 μm Sartorius Minisart filters (Satorius stedim Biotech) into 15 ml centrifuge tubes for nutrient analysis. All samples were immediately transported back to the laboratory on ice.

### Nutrient Analysis

Concentrations of ammonium ion, phosphate, nitrite, and total nitrogen were determined using a colorimetric analysis with the Lachat Quikchem QC8500 Automated Ion Analyser (LACHAT Instruments, USA) in accordance with the manufacturer’s guidelines. The resulting data were interpreted using Omnion version 3 software (LACHAT Instruments, USA).

### DNA Extraction

Microbial DNA was retrieved by filtering triplicate 2 L samples through 0.22 μm pore-size GP Sterivex membrane filters (Millipore) using a peristaltic pump. Filters were stored at -80°C until genomic DNA extraction was performed using the Powerwater DNA isolation Kit (MoBio Laboratoties, Inc.) in accordance with the manufacturer’s instructions, with the following minor modifications: (i) before extraction membrane filters were removed using sterilized scalpels and transferred into the bead tube and (ii) prior to the initial step of bead-beating and chemical lysis of cells, samples were heated to 60°C for 10 min to further aid cell lysis. DNA quantity and purity was evaluated using a Nanodrop-1000 Spectrophotometer.

### Quantitative PCR (qPCR)

Quantitative PCR (qPCR) was used to track patterns in the abundance of the total *Vibrio* community as well as pathogenic *V. cholerae, V. vulnificus*, and *V. parahaemolyticus*. qPCR was performed using the StepOnePlus^TM^ Real-Time PCR System (Applied Biosystems) and StepOne software version 2.3. All qPCR tests were run using three technical replicates, consisting of 20 μl reaction volumes containing 10 μl SYBR Select Master Mix, 3.4 μl nuclease free water, 0.4 μM for each forward and reverse primers and 5 μl of diluted (1:5) DNA template. Calibration curves were run with every plate and all extracted DNA was diluted fivefold to reduce pipetting errors.

To quantify patterns in the whole *Vibrio* community, the primer pair Vib1-f and Vib2-r (Thompson J.R. et al., 2004; Vezzulli et al., 2012) were used to amplify 16S rRNA genes specific to the *Vibrio* genus (**Table 1**). The qPCR cycling parameters involved: initial activation steps at 50°C for 120 s and 95°C for 120 s, followed by 40 cycles of a 2-step reaction involving denaturation at 95°C for 15 s and annealing/extension step at 60°C for 60 s. The amplification was concluded with a holding stage at 72°C for 120 s. To confirm that each primer pair produced only a single specific product, a melting curve was added to the end of every qPCR assay at every run (for all primers).

**Table 1:** Primers for Q-PCR detection of *Vibrio* species and for next generation sequencing.

Organism(s)	Target gene	Primers	Information on target gene	Reference
*Vibrio* genus	16S rRNA	Vib1-f (5′-GGCGTAAAGCGCATGCAGGT-3′) Vib2-r (5′-GAAATTCTACCCCCCTCTACAG-3′)	General *Vibrio* spp. (205-bp)	Thompson J.R. et al., 2004; Vezzulli et al., 2012
*Vibrio cholerae*	*ompW ctxA*	OmpW-F (5′-AACATCCGTGGATTTGGCATCTG-3′) OmpW-R (5′-GCTGGTTCCTCAACGCTTCTG-3′) ctxA_F (5′-TTTGTTAGGCACGATGATGGAT-3′) ctxA-R (5′-ACCAGACAATATAGTTTGACCCACTAAG-3′)	Species specific outer membrane protein (89-bp) Cholera toxin from toxigenic *V.cholerae* (84-bp)	Gubala, 2006; Gubala and Proll, 2006; Blackstone et al., 2007
*Vibrio vulnificus*	*vvhA*	Vul-F-1085 (5′-GGTTGCGGGTGGTTCGGT-3′) Vul-R-b1375 (5′-GATTTGCTTCATTTTCAGGGG-3′).	Hemolysin A gene from pathogenic *V. vulnificus* (290-bp)	This study
*Vibrio parahaemolyticus*	*tdhS*	TDH-169 (5′-GTAAAGGTCTCTGACTTTTGGAC-3′) TDH-415 (5′-TGGAATATGAACCTTCATCTTCACC-3′)	Thermostable direct haemolysin gene from pathogenic *V. parahaemolyticus* (270 bp)	Rizvi and Bej, 2010
*Vibrio* Genus for MiSeq Illumina platform	*16S rRNA*	Vib-169F (5′-GGATAACC/TATTGGAAACGATG-3′) Vib2-r (5′-GAAATTCTACCCCCCTCTACAG-3′)	General *Vibrio* spp. (511-bp)	Thompson J.R. et al., 2004; Yong et al., 2006; Vezzulli et al., 2012

To quantify patterns in *V. cholerae* abundance we targeted the outer-membrane protein *ompW* gene (Gubala, 2006; Gubala and Proll, 2006; **Table 1**). qPCR cycling parameters were identical to the *Vibrio* 16S rRNA assay described above, with the exception of the annealing/extension step, which occurred at 61°C for 60 s. To test for the occurrence of toxigenic *V. cholerae*, we targeted the enterotoxin gene *ctxA* (Blackstone et al., 2007; **Table 1**), using the same cycling conditions employed for the *ompW* assay.

To quantify *V. vulnificus* we targeted the hemolysin A *vvhA* gene (Panicker et al., 2004; Panicker and Bej, 2005) using a new set of primers designed for this study (**Table 1**). The *vvhA* primers were designed using Primer3 software (Thornton and Basu, 2011) and assessed against available databases with cycling conditions identical to the general *Vibrio* 16S rRNA assay.

To specifically target *V. parahaemolyticus*, the thermostable direct haemolysin *tdhS* gene was targeted (Rizvi and Bej, 2010; **Table 1**). Cycling conditions for this assay involved initial activation steps at 50°C for 120 s and 95°C for 120 s, followed by 40 cycles of a 3-step reaction incorporating: denaturation at 95°C for 15 s, annealing at 56°C for 15 s and extension step at 72°C for 60 s. The amplification was concluded with a holding stage of 72°C for 120 s followed by a melting curve. Standard curves for each qPCR assay were prepared using cultures of *V. cholerae* (O1 El Tor N16961), *V. parahaemolyticus* (ATCC17802), and *V. vulnificus* (C71840), with *V. parahaemolyticus* (ATCC17802) genomic DNA used for the *Vibrio* community 16S rRNA assay. Each strain was inoculated into a 50 ml centrifuge tube containing 6 ml of 100% marine broth (Difco Marine Broth 2216) and incubated overnight at 37°C, while shaking at 150 rpm. Samples were homogenized by vortexing and each tube was sub-sampled (1 ml × 4) into four sterile 1.5 ml tubes, creating four replicates for each *Vibrio* spp. The media was washed three times with phosphate buffer solution (PBS × 3) and centrifuged at 5200 × *g* at 10°C for 10 min. One replicate (pellet + 1 ml PBS × 3) from each bacterial strain was serially diluted and enumerated by spread plating onto triplicate marine agar plates (Difco marine Broth 2216 and +1.5% Bacteriological agar) and incubating at 37°C overnight. DNA was extracted from the other three replicates for each *Vibrio* strain using the Powerwater DNA isolation Kit (MoBio Laboratories, Inc.) in accordance with the manufacturer’s instructions. The extracted DNA (*n* = 3) was pooled and used for the generation of standard curves.

### *Vibrio* Diversity and Phylogenetic Analysis

To track shifts in the overall composition of the *Vibrio* community we combined the *Vibrio*-specific 16S rRNA primers Vib-169F (Yong et al., 2006) and Vib2-r (Thompson J.R. et al., 2004; Vezzulli et al., 2012; **Table 1**) to target the variable regions V2, V3, and part of V4. PCR reactions were performed in 50 μl volumes containing 25 μl of Mangomix^TM^ (Bioline), 0.4 μM of each of the forward and reverse primers and 1–5 μl of template DNA. PCR cycling conditions involved an initial activation step of 95°C for 120 s, followed by 30 cycles of: denaturation at 95°C for 15 s, annealing at 53°C for 30 s and extension at 72°C for 30 s, followed by a holding stage at 72°C for 10 min. After confirming positive amplification, the genomic DNA from the March and June samples was used to prepare DNA libraries with the Illumina TruSeq DNA library preparation protocol. Sequencing was performed on the Illumina MiSeq platform (at Molecular Research LP; Shallowater, TX, USA) following the manufacturer’s guidelines. Raw data files in FASTQ format were deposited in NCBI Sequence Read Archive (SRA) with the study accession number SRP069796 under Bioproject number PRJNA309925.

*Vibrio* 16S rRNA gene sequences were analyzed using the QIIME pipeline (Caporaso et al., 2010; Kuczynski et al., 2012). Briefly, paired-end DNA sequences were joined, *de novo* OTUs were defined at 97% sequence identity using UCLUST (Edgar, 2010) and taxonomy was assigned against the Greengenes database (version 13/8/2013) using BLAST (Altschul et al., 1990). Chimeric sequences were detected using ChimeraSlayer (Haas et al., 2011) and filtered from the dataset. Sequences were then rarefied to the same depth to remove the effect of sampling effort upon analysis.

### Statistical Analysis

Seasonal differences in environmental parameters were tested with One-way ANOVA followed by Tukey HSD test, performed using STATISTICA version 10.0 (StatSoft, Tulsa, OK, USA). To compare total *Vibrio* spp. abundance, data was log| x + 1| transformed and a factorial ANOVA followed by Tukey HSD test was performed using STATISTICA version 10.0 (StatSoft, Tulsa, OK, USA). Data from the *V. cholerae, V. parahaemolyticus*, and *V. vulnificus* qPCR assays were analyzed using the Kruskal–Wallis (non-parametric) test, followed by a Multiple Comparisons Assessment (StatSoft, Tulsa, OK, USA). Correlations between qPCR results and environmental parameters were assessed using Minitab 17 (Minitab Inc.). For the *Vibrio* specific amplicon sequencing data, a non-metric MDS analysis was performed on the top 50 OTUs and normalized [(x-mean)/stdev] environmental parameters, with the Bray–Curtis similarity measurement using PAST (Hammer et al., 2001). In addition, PAST was also used for SIMPER analysis, with the Bray–Curtis similarity measurement used to identify the OTUs that contributed the most to differences between the winter and late summer samples. Alpha diversity parameters of the rarefied sequences and Jackknife Comparison of the weighted sequence data were calculated in QIIME (Caporaso et al., 2010; Kuczynski et al., 2012). Differences in the alpha diversity parameters were tested using the non-parametric Mann-Whitney *U* Test (StatSoft, Tulsa, OK, USA).

## Results

### Environmental Conditions

The environmental parameters measured during the study period are summarized in **Supplementary Table S1**. Water temperature, DO and pH varied significantly between seasons [ANOVA, *F*_(4,16)_ = 115.3, *p* < 0.0001], with mean water temperatures decreasing by 8.8°C degrees (Tukey HSD, *p* < 0.0002), DO increasing by 1.6 mg L^-1^ (*p* < 0.0004) and pH increasing by 0.55 (Tukey HSD, *p* < 0.0021) from the summer to winter sampling periods. During summer, salinity decreased across the estuary from the two most eastern sites at Chowder Bay and Mort Bay, where salinities were near to marine conditions, to the western sites including Olympic Park, Rhodes, and Parramatta Park, where salinity dropped to 0–14.1 ppt. Olympic Park and Rhodes displayed the highest NH_4_^+^ levels during the winter (485.3 ± 105.3, 510.1 ± 192.6; average ± stdev μg L^-1^) while Olympic Park and Parramatta Park displayed the highest NH_4_^+^ levels during the summer (313 ± 23.8, 125.9 ± 13.1 μg L^-1^).

### Total *Vibrio* spp. Abundance

**FIGURE 2:**
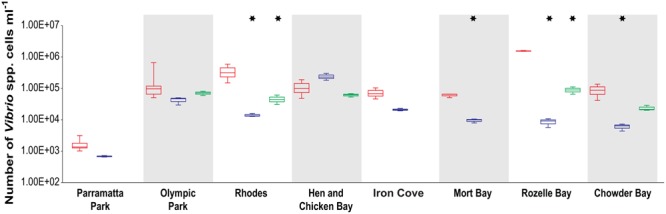
**Total *Vibrio* abundance (mL^-1^) in Sydney Harbour determined by quantitative PCR.** Red boxplots denote summer (March) samples, and blue and green boxplots denote winter [June and August (only at five location), respectively] samples. Asterisks indicate a significant difference between summer–winter samples.

Quantitative PCR analysis of the total *Vibrio* community (**Figure 2**) revealed significant differences between locations [ANOVA, *F*_(4,48)_ = 19.4, *p* < 0.0001] and sampling periods [ANOVA, *F*_(1,48)_ = 228, *p* < 0.0001], as well as a significant interactive effect between location and sampling periods [ANOVA, *F*_(11,48)_ = 17.3, *p* < 0.0001]. Most locations (seven out of eight) exhibited higher *Vibrio* spp. abundances during the summer (March) than the winter (June and/or August) with five sites (Rhodes, Mort Bay, Rozelle Bay, and Chowder Bay) displaying significant differences between summer and winter (Tukey HSD, *p* < 0.05, asterisks in **Figure 2**). Mean abundances across the entire estuary were 2.6 × 10^5^ cells ml^-1^ in the summer, compared to 4.5 × 10^4^ cells ml^-1^ in the winter. The highest concentrations were measured in Rozelle Bay during the summer (1.6 × 10^6^ cells ml^-1^) while the lowest concentration was recorded in Parramatta Park during the winter (6.5 × 10^2^ cells ml^-1^).

Across the entire data set, total *Vibrio* spp. abundance demonstrated a positive correlation to temperature (*r* = 0.306, *p* = 0.011) and a negative correlation (*r* = -0.287, *p* = 0.017) to DO. Within sites, total *Vibrio* abundance was positively correlated to temperature (*r* ≥ 0.8, *p* < 0.05) and negatively correlated to pH (*r* ≤ -0.79, *p* ≤ 0.038) at Chowder Bay, Mort Bay, Rozelle Bay, Iron Cove and Rhodes, while it was negatively correlated to salinity (*r* ≥ 0.83, *p* < 0.05) at Iron Cove and Mort Bay.

### Abundance of Pathogenic Vibrios

**FIGURE 3:**
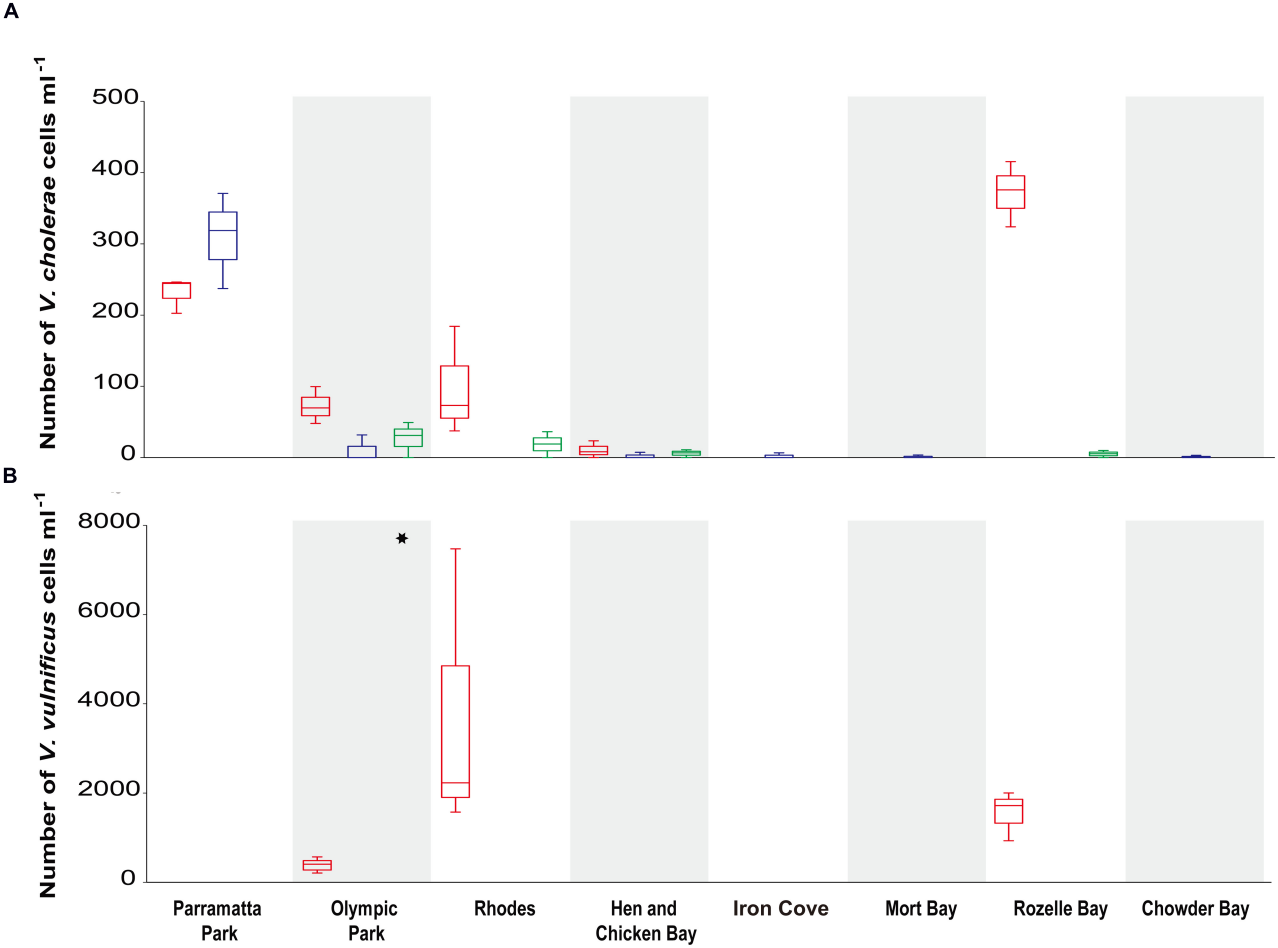
***Vibrio cholerae* and *V. vulnificus* abundance (mL^-1^) in Sydney Harbour determined by quantitative PCR.** Specific qPCR assays were carried out on: **(A)** the *V. cholerae* outer-membrane protein gene *ompW* (Efficiency ≥ 93.3% and *R*^2^ ≥ 0.996; top graph) and for **(B)** the *V. vulnificus* hemolysin A *vvhA* gene (Efficiency = 92.8% and *R*^2^ = 0.995; bottom graph). Red boxplots denote summer (March) samples, and blue and green boxplots denote winter [June and August, respectively] samples. Asterisks indicate a significant difference in *V. vulnificus* between sample locations.

*Vibrio cholerae* was detected in the majority (71%) of samples when we targeted the species specific *ompW* gene, but levels of toxigenic *V. cholerae* (positive for *ctxA*) were below detection limit in all samples. In three out of the four sites with the highest *V. cholerae* abundance (Olympic Park, Rhodes, and Rozelle Bay), summer concentrations (72 ± 26, 98 ± 76, 372 ± 46; cells ml^-1^ average ± stdev) were higher than winter concentrations. Significant differences in *V. cholerae* abundance were observed between sites [Kruskal–Wallis test: H_(7,69)_ = 15.5, *p* = 0.0297], with the highest numbers recorded at Rozelle Bay during the late summer, where concentrations reached 3.7 × 10^2^ ± 46 (average ± stdev) cells ml^-1^ (**Figure 3**).

Across the entire data set, *V. cholerae* abundance was negatively correlated to DO (*r* < -0.428, *p* < 0.0001) and to pH [*r* < -0.328, *p* < 0.006]. The nature of correlations between *V. cholerae* abundance and environmental parameters observed over time varied significantly between locations, with three sites in particular characterized by significant correlations. Within Rozelle Bay, *V. cholerae* abundance was negatively correlated to DO, pH, and NH_4_^+^, but positively correlated to temperature (*r* ≥ 0.89, *p* < 0.005). Similarly, in Rhodes, *V. cholerae* abundance was negatively correlated to DO and pH, but positively correlated to temperature (*r* ≥ 0.75, *p* < 0.02). On the other hand in Parramatta Park, *V. cholerae* abundance was negatively correlated to DO, temperature and NO_2_, but positively correlated to pH and salinity (*r* ≥ 0.73, *p* < 0.05).

*Vibrio vulnificus* was detected in 14% of all samples (**Figure 3**), but was only observed during summer and at sites within the salinity range of 5–26 ppt (**Supplementary Table S1**). There were significant differences in *V. vulnificus* abundance between locations [Kruskal–Wallis test: *H*_(2,12)_ = 8.6, *p* = 0.0134], with the highest concentration and variation (3.6 × 10^4^ ± 3 × 10^4^ cells ml^-1^; average ± stdev) recorded at Rhodes (**Figure 3**). *V. parahaemolyticus* levels were below detection limit in all samples.

### *Vibrio* Community Diversity and Structure

**FIGURE 4:**
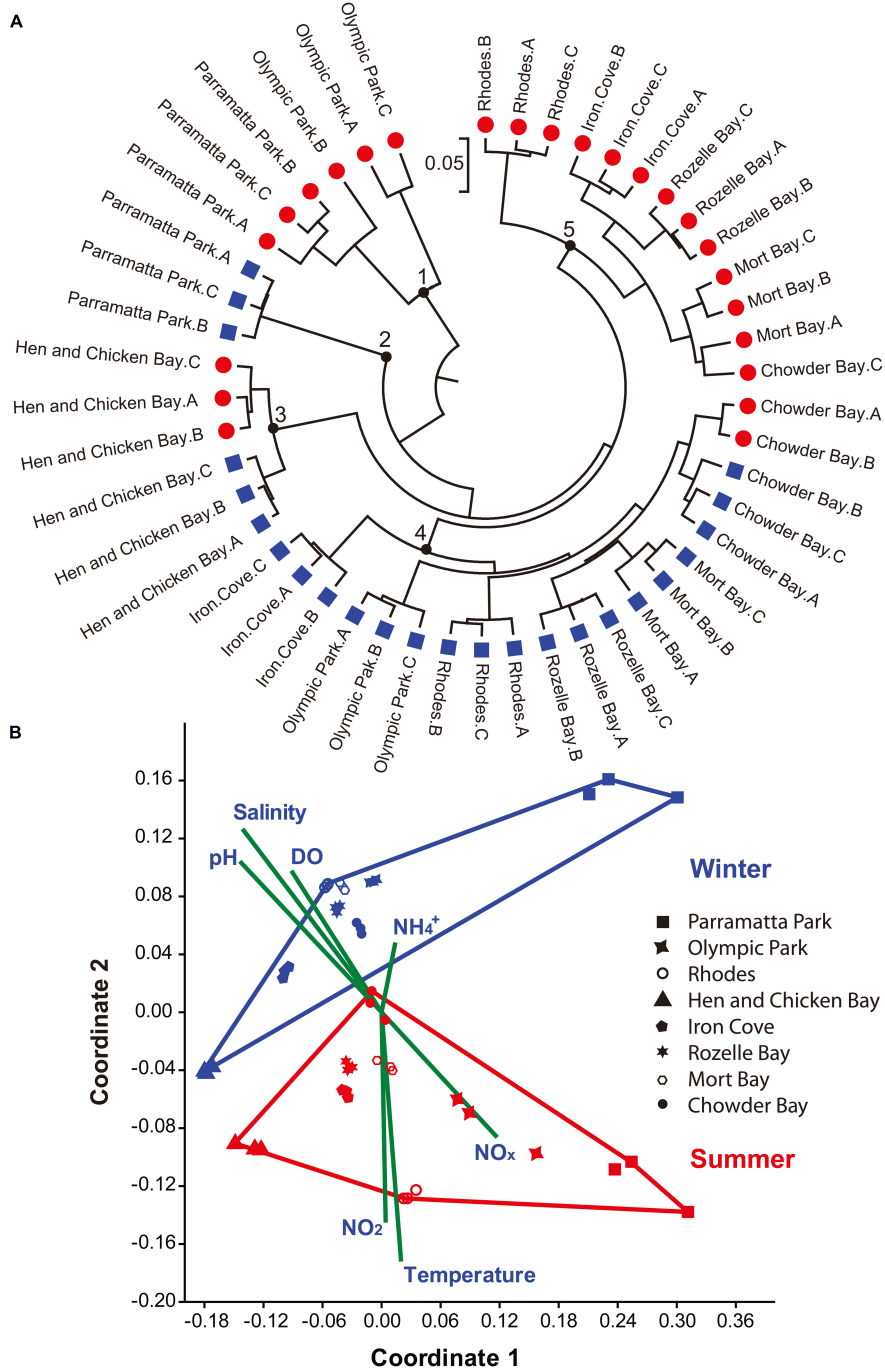
**Sample assessment using 16S rRNA gene sequences and environmental parameters. (A)** Jackknife Comparison on the weighted sequence data was carried out using QIIME (Caporaso et al., 2010) and constructed with MEGA 6.0 (Tamura et al., 2013). Red circles denote summer (March) and Blue squares denote winter (June) samples. The bar represents five substitutions per 100 nucleotide positions. Black circles numbered 1–5 highlight different groups of sequences. **(B)** Non-metric MDS plot of the top 50 OTUs. Red and Blue points represent summer and winter samples, respectively. A, B, and C represent the three biological replicates. Shepard plot stress value was 0.1237. Vectors indicate the contribution of each environmental parameter using PAST (Hammer et al., 2001).

**FIGURE 5:**
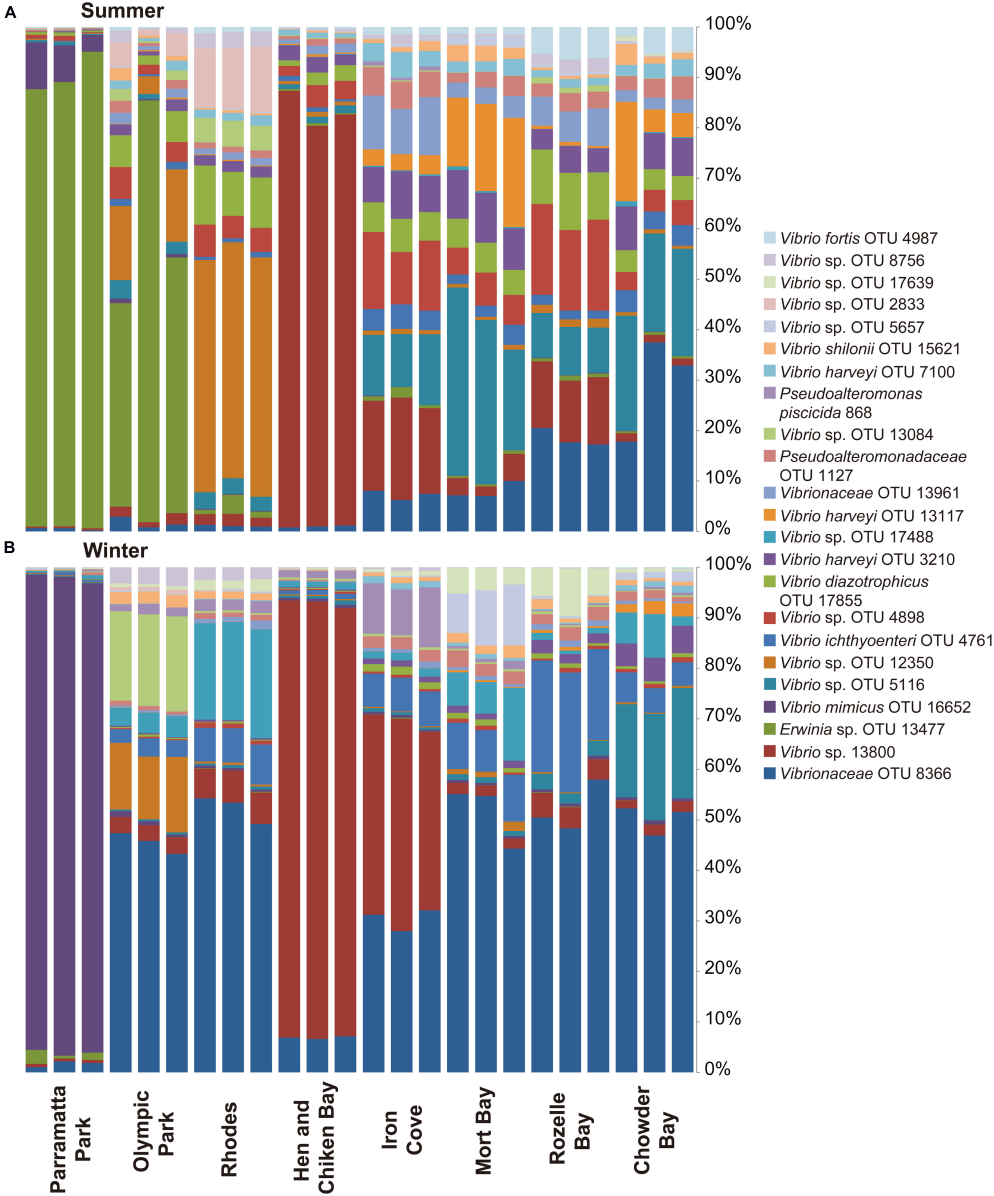
**Most abundant *Vibrio* OTUs (representing 67.5% of the total normalized sequences during winter and summer.** The figure displays the distribution of OTUs (for graphical reasons only OTUs that represent >0.5% of the total sequences were included) at the different sites, ranging from Parramatta Park in the west to Chowder Bay in the east: **(A)** summer sampling time (March), **(B)** winter (June).

Sequences were rarefied to 10,734 sequences per sample, to remove the effect of sampling effort upon analysis. Jackknife Comparison of the weighted 16S rRNA data sequences separated the sequence data into five different groups, (**Figure 4A**). In most (87%) locations and during most sampling times all three biological replicates grouped together, with a clear separation between summer and winter samples observed across the data set (**Figure 4A**). However, at two sites (Hen and Chicken Bay and Chowder Bay) the summer and winter samples grouped together (groups 3 and 4; **Figure 4A**). A non-metric MDS analysis of the top 50 OTUs, plotted together with normalized environmental parameters (representing 75.9% of the total rarefied sequence; Stress = 0.1237; **Figure 4B**) demonstrated clear divisions between summer and winter samples. However, it is notable that the three eastern most sampling sites (Chowder Bay, Rozelle Bay, and Mort Bay) displayed substantially more similarity across the seasons than the more western sampling sites (**Figures 4B** and **5**), implying that seasonal shifts were more dramatic in the western region of the estuary. Temperature and salinity were the two most significant environmental drivers of differences in the *Vibrio* community between summer and winter (**Supplementary Table S1**, **Figure 4B**). The higher summer temperatures coincided with lower salinity, DO and pH levels (**Supplementary Table S1**, **Figure 4B**).

Both spatial and temporal shifts in the composition of the *Vibrio* community were observed (**Figures 4** and **5**), with the majority (61%) of the most abundant OTUs (including all OTUs representing >0.5% of the sequences, which together comprised 67.5% of total sequences) observed during summer. However, it is notable that these dominant OTUs decreased in their relative importance during the winter (**Figure 5**). Moreover, Shannon Wiener index, observed species and species richness (Chao1), were all significantly higher in summer than winter (Mann–Whitney *U* Test, *p* ≤ 0.012). SIMPER analysis revealed that 4 OTUs, including OTU 8366 (16.5% contribution to the difference between winter and summer in **Figure 4B**), OTU 13800 (14.7%), OTU 13477 (12.4%), and OTU 16652 (8.8%), both represented the most abundant sequences in the data-set and together contributed 52.4% of the difference between winter and summer *Vibrio* communities. The Greengenes analysis assigned these sequences as follows: OTU 13800 – to the genus *Vibrio*, OTU 8366 – to the *Vibrionaceae* family, OTU 13477 – to the genus *Erwinia* and OTU 16652 – to *V. mimicus*.

## Discussion

Our study revealed that *Vibrios*, including several human pathogens, are an abundant and dynamic component within the Sydney Harbour estuary, and that the community displays marked spatiotemporal heterogeneity that can often be clearly linked to environmental variables including temperature. Temperature significantly influenced both *Vibrio* abundance and community composition within Sydney Harbour, with highest concentrations observed during the warmer summer sampling period. This is consistent with evidence that many *Vibrio* species prefer warmer waters and is in-line with the seasonal dynamics observed in several other regions including the North Sea, Tyrrhenian coast brackish water, coastal regions of the Northern Baltic and along the east coast of the USA (Eiler et al., 2007; Turner et al., 2009; Caburlotto et al., 2012; Oberbeckmann et al., 2012; Vezzulli et al., 2012; Takemura et al., 2014; Matteucci et al., 2015). Indeed in other coastal regions it has been demonstrated that water temperature and salinity are the major drivers of patterns in *Vibrio* abundance (Oberbeckmann et al., 2012; Jacobs et al., 2014; Takemura et al., 2014).

While temperature was the most significant environmental determinant across the entire data-set, within sampling-periods and individual sites salinity was a key environmental determinant of patterns in *Vibrio* abundance and diversity. Negative correlations between *Vibrio* abundance and salinity were observed in two of the most easterly and ‘marine’ sites (Iron Cove and Mort Bay), while at the most western and fresh water site at Parramatta Park a positive correlation between salinity and *V. cholerae* was observed. Whereas, *V. vulnificus* occurred within the salinity range of 5–26 ppt (Olympic Park, Rhodes, and Rozelle Bay) supporting previous observations that salinity optimum varies between different *Vibrio* species (Kaspar and Tamplin, 1993; Randa et al., 2004; Hsieh et al., 2008; Takemura et al., 2014).

### Presence of Pathogenic *Vibrios* in Sydney Harbour

We targeted three pathogenic *Vibrio* spp. known to have substantial relevance to human health. Several outbreaks of pathogenic *Vibrios* have previously been documented in Australia. In the 1970s, a small outbreak of cholera occurred in south-eastern Queensland (Rao and Stockwell, 1980), which was linked to local riverine toxigenic *V. cholerae* populations (Rao and Stockwell, 1980; Rogers et al., 1980), and the presence of toxigenic and non-toxigenic *V. cholerae* has subsequently been documented within several rivers and estuaries in eastern Australia (Desmarchelier and Reichelt, 1981; Bourke et al., 1986; Desmarchelier et al., 1988, 1995; Mallard and Desmarchelier, 1995; Islam et al., 2013). Other pathogenic *Vibrios* and *Vibrio* spp. related infections have been reported in Australia. Isolates of *V. cholerae* and *V. parahaemolyticus* have been extracted from *Crassostrea commercialis* (Sydney rock oysters) farmed at estuaries along the east coast of Australia (Eyles et al., 1985; Eyles and Davey, 1988) and several cases of *V. vulnificus* and *V. parahaemolyticus* infections of humans have been reported from estuaries in Northern Australia, following skin contact with river water and seawater (Ralph and Currie, 2007).

In our study, *V. cholerae* was detected at most sites within Sydney Harbour, with highest abundances observed during late summer, at sites with low to mid-range salinity levels (Parramatta Park, Olympic Park, Rhodes, and Rozelle Bay; 0–26 ppt). Similarly, temporal studies in another urban estuary along the Australian east coast identified the highest prevalence of *V cholerae* in water, sediment and in oysters during March–May (Eyles and Davey, 1988). It is possible that this high abundance of *V cholerae* in warm brackish waters followed an increase of dissolved organic matter following a phytoplankton bloom (Eiler et al., 2007). However, it is important to note that not all strains of *V. cholerae* are responsible for causing cholera, indeed only the serogroups O1/O139 are responsible. The *ompW* gene used here differentiates *V. cholerae* from other *Vibrios*, but does not necessarily identify disease causing strains of *V. cholerae.* Our subsequent analysis targeting the *ctxA* (cholera toxin) gene did not detect this gene in any samples, suggesting that during our study, toxigenic *V. cholerae*, which is the causative agent of cholera (Thompson F.L. et al., 2004; Nelson et al., 2009), was not present in the water of Sydney Harbour estuary. However, other, non-toxigenic strains of *V. cholerae* are also responsible for severe skin infections (Andersson and Ekdahl, 2006; Lukinmaa et al., 2006; Stypulkowska-Misiurewicz et al., 2006) and gastroenteritis (Dutta et al., 2013; Luo et al., 2013). Additionally, non-toxigenic *V. cholerae* strains from Sydney, that lack the *ctxA* gene, have been shown to be pathogenic through Rabbit Ileal Loop models, where pathogenesis (fluid accumulation) occurred due to the presence of other virulence factors (Islam et al., 2013). The presence of these alternate virulence factors in non-O1/non-O139 *V. cholerae* isolates has been observed in Iceland (Haley et al., 2012) and in the Botany Bay catchment (Islam et al., 2013), which is immediately adjacent to Sydney Harbour. Therefore, while not necessarily involved in cholera, the strains of *V. cholerae* identified here are still likely to be significant within the context of human health. Moreover, since *Vibrios* are particularly prone to lateral gene transfer, non-toxigenic strains of *V. cholerae* can acquire virulence through horizontal gene transfer especially following cellular interactions within localized microenvironments such as on zooplankton and within biofilms (Borgeaud et al., 2015; Metzger and Blokesch, 2016).

The links between *V. cholerae* abundance and season and temperature observed here are significant, because elsewhere warming patterns have coincided with outbreaks of *Vibrio* infections (including infections from non-toxigenic *V. cholerae*), with the distribution of infection cases closely corresponding to the temporal and spatial peaks in sea surface temperatures (Baker-Austin et al., 2013; Sterk et al., 2015). In regions where cholera occurs, there are also clear links between elevated temperatures and cholera outbreaks (Huq et al., 2005; Colwell, 2009).

The detection of the virulence gene *vvhA* by qPCR confirmed the presence of pathogenic *V. vulnificus* populations in three of the tested sites within the Sydney Harbour catchment during the late summer (March) sampling session. However, this organism was not detected at any sites during the winter sampling. This is consistent with patterns observed elsewhere, whereby the abundance of this species is generally correlated with elevated water temperatures (Jacobs et al., 2014), with the occurrence of *V. vulnificus* often limited to the summer months (Randa et al., 2004). As a consequence, highest frequencies of human infections typically occur during the warmer summer months (Strom and Paranjpye, 2000). The three sites where *V. vulnificus* was detected are located mid-estuary, where salinity levels were between 5 and 26 ppt. This also falls within the salinity range where *V. vulnificus* abundance has been shown to be highest elsewhere (Kaspar and Tamplin, 1993; Randa et al., 2004).

It has been suggested that increased *V. vulnificus* abundance within marine environments increases both the risk of infection from seafood consumption (Strom and Paranjpye, 2000; Chu et al., 2011) and direct exposure from swimming (Daniels and Shafaie, 2000). The highest concentration of *V. vulnificus* observed in this study was 7 × 10^4^ cells ml^-1^. Whether these levels are sufficient to induce infection in humans is unclear since the infectious dose of this organism is currently unknown and probably dependent on host factors (Daniels and Shafaie, 2000). However, the high fatality rate associated with *V. vulnificus* infection (Daniels and Shafaie, 2000; Jones and Oliver, 2009; Horseman and Surani, 2011; Ralston et al., 2011) warrants a level of concern regarding the occurrence of this bacterium within the heavily used waters of Sydney Harbour.

We also expected to detect pathogenic *V. parahaemolyticus* within Sydney Harbour, at least during the summer, since this species is commonly found in estuarine waters above 20°C (Nigro et al., 2011; Matteucci et al., 2015) and has previously been identified in Sydney rock oysters (Eyles et al., 1985). However, in all samples, *V. parahaemolyticus* levels were below the detection limit of our qPCR assay. It is important to emphasize that we tested only the water while other studies detected these bacteria within oysters or associated with other marine animals (Eyles and Davey, 1984; Eyles et al., 1985). Moreover, given the high levels of spatiotemporal variability observed in our study, this finding does not eliminate the possibility that these bacteria occur in the Sydney Harbour catchment water at other times.

### Patterns in *Vibrio* Community Diversity and Composition

In addition to the shifts in abundance described above, we observed substantial spatiotemporal changes in the composition of the *Vibrio* community within Sydney Harbour. Unfortunately, in some OTU’s the assigned taxonomy was above species and genus level due to the 16S rRNA gene’s limited resolution in the *Vibrionaceae* family (Sawabe et al., 2013). *Vibrio* diversity, as determined by our *Vibrio*-specific 16S rRNA gene sequencing, was higher in late summer than winter. Furthermore, *Vibrio* community composition was clearly separated into winter and summer groups within an MDS plot (**Figure 4B**). Analysis of the sequences without the inclusion of environmental parameters was not as conclusive (**Figure 4A**). While sequences from six of the locations separated between summer and winter groups, in two locations: Hen and Chicken Bay and Chowder Bay different patterns were observed. In Hen and Chicken Bay, the winter and summer samples grouped together, but separately from all other sites. However, in Chowder Bay individual replicates from the summer sampling period were divided between the winter and summer groups.

The eastern-most site, closest to the open ocean, at Chowder Bay, displayed the lowest seasonal variability in salinity, temperature and DO (**Supplementary Table S1**), which may explain the inter-seasonal consistency in *Vibrio* community composition at this site. The *Vibrio* community at Hen and Chicken Bay was universally separated from all other sites, which could be explained by the fact that this was the only sampling site where samples were obtained from relatively shallow water (∼1 m) leading to a potentially greater influence from resuspended sediments. We analyzed patterns in the *Vibrio* community collected on a 0.22 μm filter, meaning that both free-living and particle-attached populations were considered together. There is evidence that a particle attached lifestyle is common for many *Vibrios* (Takemura et al., 2014), and although data on suspended particle concentrations were not collected during this study it remains possible that some of the spatiotemporal patterns observed here were underpinned by variability in the concentrations of suspended particulate material in the estuary.

In accordance with the patterns in total *Vibrio* abundance, temperature was one of the most significant drivers of differences in the *Vibrio* community composition. This pattern supports previous observations that have indicated that elevated water temperature is a major factor explaining patterns in *Vibrio* community composition and abundance (Nishiguchi, 2000; Thompson J.R. et al., 2004; Tout et al., 2015).

Some of the differences in *Vibrio* abundance between the winter samples might be explained by differential influence of a rainfall event, where 58.3 mm of rain occurred during the week proceeding August samples collection (Observatory Hill station, Sydney, http://www.bom.gov.au/climate/data/index.shtml). Stormwater run-off associated with rainfall events influence salinity and nutrient levels within Sydney Harbour, which can drive heterogeneity in bacterioplankton community composition within the estuary (Jeffries et al., 2015). During each sampling period, and particularly during March (summer sample), spatial shifts in the composition of the *Vibrio* community were primarily governed by variability in salinity, which is consistent with patterns previously observed in other environments (Oberbeckmann et al., 2012; Jacobs et al., 2014; Takemura et al., 2014). While temperature and salinity were the most important drivers of shifts in overall *Vibrio* community structure, and total *Vibrio* abundance, not all *Vibrio* spp. displayed the same patterns. For instance, the relative proportion of the OTU (16652) with closest sequence matches to *V. mimicus*, and which comprised up to 10% of the community in three samples, increased significantly during winter in the Parramatta Park samples. This is consistent with observations in Bangladesh, where *V. mimicus* numbers spiked during lower temperature months (Chowdhury et al., 1989). This is particularly notable because *V. mimicus* has been implicated in outbreaks of vibriosis in freshwater catfish in China, leading to 80–100% mortality rates (Geng et al., 2014). *V. mimicus* is also pathogenic to humans, causing diarrheal disease (Hlady and Klontz, 1996; Adebayo-Tayo et al., 2011), with some isolates exhibiting the presence of the lysogenic filamentous bacteriophage that carries the cholera toxin genes in epidemic *V. cholerae* strains (Boyd et al., 2000). So the substantial proportion of *Vibrio* sequences matching this organism within regions of the Sydney Harbour estuary is also noteworthy.

### Environmental and Human Health Implications

Previous studies that have examined *Vibrio* related infections occurring in coastal sites in the Baltic Sea, North Sea, Israel, and the Korean peninsula have revealed the link between high water temperatures and infection rates (Paz et al., 2007; Hashizume et al., 2008; Chu et al., 2011; Baker-Austin et al., 2013). Furthermore, recent models have revealed that elevated water temperature is a key factor explaining *Vibrio* abundance within aquatic samples (Oberbeckmann et al., 2012; Jacobs et al., 2014). These patterns and predictions are relevant within the context of the current, and predicted future, warming of seawater temperatures along the south-eastern Australian coast (Cai et al., 2005; Hobday and Lough, 2011; Last et al., 2011). We performed a simple analysis of surface seawater temperatures (SST) along the Sydney coastline (34°05′S 151°15′E) during the last 57 years (**Supplementary Figure S1**), which suggests that a rise in SST of 1–2°C will increase the number of days where SST ≥ 20°C, which is the preferred temperature regime of several pathogenic *Vibrio* species (Takemura et al., 2014), by up to 169%. This is particularly relevant within the context of recent evidence for temperature-induced shifts in bacterial community composition and function in this region (Seymour et al., 2012), and a precedent of previous *Vibrio* outbreaks within the waters of eastern Australia (Rao and Stockwell, 1980; Rogers et al., 1980; Desmarchelier et al., 1995).

Developing an understanding of the spatiotemporal dynamics of *Vibrio* populations and identifying key environmental drivers are essential for predicting future risks and hotspots for pathogen outbreaks within heavily used coastal ecosystems such as Sydney Harbour. Our observations suggest that several sites within Sydney Harbour, where significant abundances of *V. cholerae* and *V. vulnificus* already occur, are potentially at risk of pathogenic *Vibrio* outbreaks, particularly during warm summer months. Moreover, since many of the sites where pathogenic *Vibrio* were identified are situated near to river inputs or adjacent to stormwater canals, the combination of warm summer conditions and the typically high summer rainfall in Sydney (which will often reduce salinity levels and add nutrients) have the potential to provide a ‘perfect storm,’ within the context of conditions favoring pathogenic *Vibrio* outbreaks.

## Author Contributions

NS and VB contributed equally to this research. NS, VB, JS, and ML conceived and designed the experiments. NS, VB, RC, and JS collected the samples. RC and VB performed the nutrient analysis. NS and VB performed the molecular analysis. NS analysed the data. NS, ML, and JS wrote the paper.

## Conflict of Interest Statement

The authors declare that the research was conducted in the absence of any commercial or financial relationships that could be construed as a potential conflict of interest.

## Supplementary Material

FIGURE S1 **Distribution of the average seawater surface temperatures at Port-Hacking**.

TABLE S1 **Sample location and environmental associated data**.

## References

[B1] Adebayo-Tayo B. Okonko I. John M. Odu N. Nwanze J. Ezediokpu M. (2011). Occurrence of potentially pathogenic *Vibrio* species in Sea foods obtained from Oron Creek. *Adv. Biol. Res.* 5 356–365.

[B2] Ali M. Lopez A. L. You Y. Kim Y. E. Sah B. Maskery B. (2012). The global burden of cholera. *Bull. World Health Organ.* 90 209–218A. 10.2471/BLT.11.093427 22461716 PMC3314202

[B3] Altschul S. F. Gish W. Miller W. Myers E. W. Lipman D. J. (1990). Basic local alignment search tool. *J. Mol. Biol.* 215 403–410. 10.1016/S0022-2836(05)80360-2 2231712

[B4] Andersson Y. Ekdahl K. (2006). Wound infections due to *Vibrio cholerae* in Sweden after swimming in the Baltic Sea, summer 2006. *Euro Surveill.* 11:E060803.2. 10.2807/esw.11.31.03013-en 16966771

[B5] Armitage N. Rooseboom A. (2000). The removal of urban litter from stormwater conduits and streams: paper 1- The quantities involved and catchment litter management options. *Water SA* 26 181–188.

[B6] Austin B. Zhang X. H. (2006). *Vibrio harveyi*: a significant pathogen of marine vertebrates and invertebrates. *Lett. Appl. Microbiol.* 43 119–124. 10.1111/j.1472-765X.2006.01989.x 16869892

[B7] Australian Bureau of Statistics (2004). *How Many People Live in Australia’s Coastal Areas? In Year Book Australia 2004 (Cat. No. 1301.0).* Canberra, ACT: Commonwealth of Australia.

[B8] Baker-Austin C. Stockley L. Rangdale R. Martinez-Urtaza J. (2010). Environmental occurrence and clinical impact of *Vibrio vulnificus* and *Vibrio parahaemolyticus*: a European perspective. *Environ. Microbiol. Rep.* 2 7–18. 10.1111/j.1758-2229.2009.00096.x 23765993

[B9] Baker-Austin C. Trinanes J. A. Taylor N. G. Hartnell R. Siitonen A. Martinez-Urtaza J. (2013). Emerging *Vibrio* risk at high latitudes in response to ocean warming. *Nat. Clim. Change* 3 73–77. 10.1038/nclimate1628

[B10] Becker P. Gillan D. Lanterbecq D. Jangoux M. Rasolofonirina R. Rakotovao J. (2004). The skin ulceration disease in cultivated juveniles of *Holothuria scabra* (Holothuroidea, Echinodermata). *Aquaculture* 242 13–30. 10.1016/j.aquaculture.2003.11.018

[B11] Ben-Haim Y. Rosenberg E. (2002). A novel *Vibrio* sp. pathogen of the coral *Pocillopora damicornis*. *Mar. Biol.* 141 47–55. 10.1007/s00227-002-0797-6

[B12] Birch G. (1996). Sediment-bound metallic contaminants in Sydney’s estuaries and adjacent offshore, Australia. *Estuar. Coast. Shelf Sci.* 42 31–44. 10.1006/ecss.1996.0003

[B13] Blackstone G. M. Nordstrom J. L. Bowen M. D. Meyer R. F. Imbro P. DePaola A. (2007). Use of a real time PCR assay for detection of the ctxA gene of *Vibrio cholerae* in an environmental survey of Mobile Bay. *J. Microbiol. Methods* 68 254–259. 10.1016/j.mimet.2006.08.006 17034889

[B14] Blackwell K. D. Oliver J. D. (2008). The ecology of *Vibrio vulnificus, Vibrio cholerae*, and *Vibrio parahaemolyticus* in North Carolina estuaries. *J. Microbiol.* 46 146–153. 10.1007/s12275-007-0216-2 18545963

[B15] Borgeaud S. Metzger L. C. Scrignari T. Blokesch M. (2015). The type VI secretion system of *Vibrio cholerae* fosters horizontal gene transfer. *Science* 347 63–67. 10.1126/science.1260064 25554784

[B16] Bourke A. Cossins Y. N. Gray B. Lunney T. J. Rostron N. A. Holmes R. V. (1986). Investigation of cholera acquired from the riverine environment in Queensland. *Med. J. Aust.* 144 229–234. 3587092 10.5694/j.1326-5377.1986.tb115883.x

[B17] Boyd E. F. Moyer K. E. Shi L. Waldor M. K. (2000). Infectious CTXΦ and the *Vibrio* pathogenicity island prophage in *Vibrio mimicus*: evidence for recent horizontal transfer between *V. mimicus* and *V. cholerae*. *Infect. Immun.* 68 1507–1513. 10678967 10.1128/iai.68.3.1507-1513.2000 PMC97308

[B18] Caburlotto G. Bianchi F. Gennari M. Ghidini V. Socal G. Aubry F. B. (2012). Integrated evaluation of environmental parameters influencing *Vibrio* occurrence in the coastal Northern Adriatic Sea (Italy) facing the Venetian lagoon. *Microb. Ecol.* 63 20–31. 10.1007/s00248-011-9920-x 21826491

[B19] Cai W. Shi G. Cowan T. Bi D. Ribbe J. (2005). The response of the Southern Annular Mode, the East Australian Current, and the southern mid-latitude ocean circulation to global warming. *Geophys. Res. Lett.* 32 1–4. 10.1029/2005GL024701

[B20] Caporaso J. G. Kuczynski J. Stombaugh J. Bittinger K. Bushman F. D. Costello E. K. (2010). QIIME allows analysis of high-throughput community sequencing data. *Nat. Methods* 7 335–336. 10.1038/nmeth.f.303 20383131 PMC3156573

[B21] Centers for Disease Control and Prevention (1998). Outbreak of *Vibrio parahaemolyticus* infections associated with eating raw oysters–Pacific Northwest, 1997. *Morb. Mortal. Wkly. Rep.* 47 457–462. 9639367

[B22] Chowdhury M. Yamanaka H. Miyoshi S. Aziz K. Shinoda S. (1989). Ecology of *Vibrio mimicus* in aquatic environments. *Appl. Environ. Microbiol.* 55 2073–2078. 2782878 10.1128/aem.55.8.2073-2078.1989 PMC203005

[B23] Chu C. Do Y. Kim Y. Saito Y. Lee S.-D. Park H. (2011). Mathematical modeling of *Vibrio vulnificus* infection in Korea and the influence of global warming. *Osong Public Health Res. Perspect.* 2 51–58. 10.1016/j.phrp.2011.05.002 24159451 PMC3766921

[B24] Colwell R. R. (2009). “Viable but not cultivable bacteria,” in *Uncultivated Microorganisms* ed. Epstein S. S. (Heidelberg: Springer) 121–129.

[B25] Daniels N. A. Shafaie A. (2000). A review of pathogenic *Vibrio* infections for clinicians. *Infect. Med.* 17 665–685.

[B26] Desmarchelier P. Momen H. Salles C. (1988). A zymovar analysis of *Vibrio cholerae* isolated in Australia. *Trans. R. Soc. Trop. Med. Hyg.* 82 914–917. 10.1016/0035-9203(88)90041-7 3256999

[B27] Desmarchelier P. Wong F. Mallard K. (1995). An epidemiological study of *Vibrio cholerae* O1 in the Australian environment based on rRNA gene polymorphisms. *Epidemiol. Infect.* 115 435–446. 10.1017/S0950268800058593 8557075 PMC2271589

[B28] Desmarchelier P. M. Reichelt J. L. (1981). Phenotypic characterization of clinical and environmental isolates of *Vibrio cholerae* from Australia. *Curr. Microbiol.* 5 123–127. 10.1128/mBio.01824-14

[B29] Ducklow H. W. Carlson C. A. (1992). “Oceanic bacterial production,” in *Advances in Microbial Ecology* ed. Marshall K. C. (New York, NY: Springer) 113–181.

[B30] Dutta D. Chowdhury G. Pazhani G. P. Guin S. Dutta S. Ghosh S. (2013). *Vibrio cholerae* non-O1 non-O139 serogroups and cholera-like diarrhea, Kolkata, India. *Emerg. Infect. Dis.* 19 464–467. 10.3201/eid1903.121156 23622872 PMC3647666

[B31] Edgar R. C. (2010). Search and clustering orders of magnitude faster than BLAST. *Bioinformatics* 26 2460–2461. 10.1093/bioinformatics/btq461 20709691

[B32] Eiler A. Gonzalez-Rey C. Allen S. Bertilsson S. (2007). Growth response of *Vibrio cholerae* and other *Vibrio* spp. to cyanobacterial dissolved organic matter and temperature in brackish water. *FEMS Microbiol. Ecol.* 60 411–418. 10.1111/j.1574-6941.2007.00303.x 17386033

[B33] Eiler A. Johansson M. Bertilsson S. (2006). Environmental influences on *Vibrio* populations in northern temperate and boreal coastal waters (Baltic and Skagerrak Seas). *Appl. Environ. Microbiol.* 72 6004–6011. 10.1128/AEM.00917-06 16957222 PMC1563599

[B34] Eyles M. Davey G. Arnold G. (1985). Behavior and incidence of *Vibrio parahaemolyticus* in Sydney rock oysters (*Crassostrea commercialis*). *Int. J. Food Microbiol.* 1 327–334. 10.1016/0168-1605(85)90004-2

[B35] Eyles M. J. Davey G. R. (1984). Microbiology of commercial depuration of the Sydney rock oyster, *Crassostrea commercialis*. *J. Food Prot.* 47 703–712. 10.4315/0362-028X-47.9.703 30934453

[B36] Eyles M. J. Davey G. R. (1988). *Vibrio cholerae* and enteric bacteria in oyster-producing areas of two urban estuaries in Australia. *Int. J. Food Microbiol.* 6 207–218. 10.1016/0168-1605(88)90013-X 3079469

[B37] Frans I. Michiels C. W. Bossier P. Willems K. Lievens B. Rediers H. (2011). *Vibrio anguillarum* as a fish pathogen: virulence factors, diagnosis and prevention. *J. Fish Dis.* 34 643–661. 10.1111/j.1365-2761.2011.01279.x 21838709

[B38] Froelich B. Bowen J. Gonzalez R. Snedeker A. Noble R. (2013). Mechanistic and statistical models of total *Vibrio* abundance in the Neuse River Estuary. *Water Res.* 47 5783–5793. 10.1016/j.watres.2013.06.050 23948561

[B39] Geng Y. Liu D. Han S. Zhou Y. Wang K. Y. Huang X. L. (2014). Outbreaks of vibriosis associated with *Vibrio mimicus* in freshwater catfish in China. *Aquaculture* 433 82–84. 10.1016/j.aquaculture.2014.05.053

[B40] Gilbert J. A. Steele J. A. Caporaso J. G. Steinbrück L. Reeder J. Temperton B. (2011). Defining seasonal marine microbial community dynamics. *ISME J.* 6 298–308. 10.1038/ismej.2011.107 21850055 PMC3260500

[B41] Goarant C. Herlin J. Brizard R. Marteau A.-L. Martin C. Martin B. (2000). Toxic factors of *Vibrio* strains pathogenic to shrimp. *Dis. Aquat. Organ.* 40 101–107. 10.3354/dao040101 10782343

[B42] González-Escalona N. Cachicas V. Acevedo C. Rioseco M. L. Vergara J. A. Cabello F. (2005). *Vibrio parahaemolyticus* diarrhea, Chile, 1998 and 2004. *Emerg. Infect. Dis.* 11 129–131. 10.3201/eid1101.040762 15705337 PMC3294363

[B43] Gubala A. J. (2006). Multiplex real-time PCR detection of *Vibrio cholerae*. *J. Microbiol. Methods* 65 278–293. 10.1016/j.mimet.2005.07.017 16153727

[B44] Gubala A. J. Proll D. F. (2006). Molecular-beacon multiplex real-time PCR assay for detection of *Vibrio cholerae*. *Appl. Environ. Microbiol.* 72 6424–6428. 10.1128/AEM.02597-05 16957277 PMC1563670

[B45] Haas B. J. Gevers D. Earl A. M. Feldgarden M. Ward D. V. Giannoukos G. (2011). Chimeric 16S rRNA sequence formation and detection in Sanger and 454-pyrosequenced PCR amplicons. *Genome Res.* 21 494–504. 10.1101/gr.112730.110 21212162 PMC3044863

[B46] Haley B. J. Chen A. Grim C. J. Clark P. Diaz C. M. Taviani E. (2012). *Vibrio cholerae* in a historically cholera-free country. *Environ. Microbiol. Rep.* 4 381–389. 10.1111/j.1758-2229.2012.00332.x 23185212 PMC3505037

[B47] Hammer Ø. Harper D. A. T. Ryan P. D. (2001). PAST-palaeontological statistics software package for education and data analysis. *Palaeontol. Electronica* 4 1–9.

[B48] Hasan N. A. Grim C. J. Lipp E. K. Rivera I. N. Chun J. Haley B. J. (2015). Deep-sea hydrothermal vent bacteria related to human pathogenic *Vibrio* species. *Proc. Natl. Acad. Sci. U.S.A.* 112 E2813–E2819. 10.1073/pnas.1503928112 25964331 PMC4450432

[B49] Hashizume M. Armstrong B. Hajat S. Wagatsuma Y. Faruque A. S. Hayashi T. (2008). The effect of rainfall on the incidence of cholera in Bangladesh. *Epidemiology* 19 103–110. 10.1097/EDE.0b013e31815c09ea 18091420

[B50] Higgins R. (2000). Bacteria and fungi of marine mammals: a review. *Can. Vet. J.* 41 105–116. 10723596 PMC1476275

[B51] Hlady W. G. Klontz K. C. (1996). The epidemiology of *Vibrio* infections in Florida, 1981–1993. *J. Infect. Dis.* 173 1176–1183. 10.1093/infdis/173.5.1176 8627070

[B52] Hobday A. J. Lough J. M. (2011). Projected climate change in Australian marine and freshwater environments. *Mar. Freshw. Res.* 62 1000–1014. 10.1016/j.marpolbul.2014.06.003

[B53] Horseman M. A. Surani S. (2011). A comprehensive review of *Vibrio vulnificus*: an important cause of severe sepsis and skin and soft-tissue infection. *Int. J. Infect. Dis.* 15 e157–e166. 10.1016/j.ijid.2010.11.003 21177133

[B54] Hsieh J. L. Fries J. S. Noble R. T. (2008). Dynamics and predictive modelling of *Vibrio* spp. in the Neuse River Estuary, North Carolina, USA. *Environ. Microbiol.* 10 57–64. 10.1111/j.1462-2920.2007.01429.x 18211266

[B55] Huq A. Sack R. B. Nizam A. Longini I. M. Nair G. B. Ali A. (2005). Critical factors influencing the occurrence of *Vibrio cholerae* in the environment of Bangladesh. *Appl. Environ. Microbiol.* 71 4645–4654. 10.1128/AEM.71.8.4645-4654.2005 16085859 PMC1183289

[B56] Hutchings P. A. Ahyong S. T. Ashcroft M. B. McGrouther M. A. Reid A. L. (2013). Sydney Harbour: its diverse biodiversity. *Aust. Zool.* 36 255–320. 10.7882/AZ.2012.031

[B57] Islam A. Labbate M. Djordjevic S. P. Alam M. Darling A. Melvold J. (2013). Indigenous *Vibrio cholerae* strains from a non-endemic region are pathogenic. *Open Biol.* 3 120181 10.1098/rsob.120181 PMC3603452 23407641

[B58] Jacobs J. M. Rhodes M. Brown C. W. Hood R. R. Leight A. Long W. (2014). Modeling and forecasting the distribution of *Vibrio vulnificus* in Chesapeake Bay. *J. Appl. Microbiol.* 117 1312–1327. 10.1111/jam.12624 25139334

[B59] Jeffries T. C. Schmitz Fontes M. L. Harrison D. P. van Dongen-Vogels V. Eyre B. D. Ralph P. J. (2015). Bacterioplankton dynamics within a large anthropogenically impacted urban estuary. *Front. Microbiol.* 6:1438 10.3389/fmicb.2015.01438 PMC4726783 26858690

[B60] Jones M. K. Oliver J. D. (2009). *Vibrio vulnificus*: disease and pathogenesis. *Infect. Immun.* 77 1723–1733. 19255188 10.1128/IAI.01046-08 PMC2681776

[B61] Kaspar C. Tamplin M. (1993). Effects of temperature and salinity on the survival of *Vibrio vulnificus* in seawater and shellfish. *Appl. Environ. Microbiol.* 59 2425–2429. 8368832 10.1128/aem.59.8.2425-2429.1993 PMC182301

[B62] Kubota K. (2015). “Estimating the burden of foodborne illness for campylobacter, salmonella and *Vibrio parahaemolyticus* in Japan, 2006–2012,” in *Proceedings of the Annual Meeting 2015 July 25–28, 2015* (Des Moines, IA: International Association for Food Protection).

[B63] Kuczynski J. Stombaugh J. Walters W. A. González A. Caporaso J. G. Knight R. (2012). Using QIIME to analyze 16S rRNA gene sequences from microbial communities. *Curr. Protoc. Bioinformatics* Chap. 10:Unit 10.7 10.1002/0471250953.bi1007s36 PMC3249058 22161565

[B64] Kushmaro A. Banin E. Loya Y. Stackebrandt E. Rosenberg E. (2001). *Vibrio shiloi* sp. nov., the causative agent of bleaching of the coral *Oculina patagonica*. *Int. J. Syst. Evol. Microbiol.* 51 1383–1388. 10.1099/00207713-51-4-1383 11491336

[B65] Last P. R. White W. T. Gledhill D. C. Hobday A. J. Brown R. Edgar G. J. (2011). Long-term shifts in abundance and distribution of a temperate fish fauna: a response to climate change and fishing practices. *Glob. Ecol. Biogeogr.* 20 58–72. 10.1111/j.1466-8238.2010.00575.x

[B66] Lee K.-H. Ruby E. G. (1994). Effect of the squid host on the abundance and distribution of symbiotic *Vibrio fischeri* in nature. *Appl. Environ. Microbiol.* 60 1565–1571. 16349257 10.1128/aem.60.5.1565-1571.1994 PMC201518

[B67] Lee S. B. Birch G. F. (2014). “Sydney estuary, australia: geology, anthropogenic development and hydrodynamic processes/attributes,” in *Estuaries of Australia in 2050 and Beyond* ed. Wolanski E. (Dordrecht: Springer) 17–30.

[B68] Lipp E. K. Huq A. Colwell R. R. (2002). Effects of global climate on infectious disease: the cholera model. *Clin. Microbiol. Rev.* 15 757–770. 10.1128/CMR.15.4.757-770.2002 12364378 PMC126864

[B69] Lukinmaa S. Mattila K. Lehtinen V. Hakkinen M. Koskela M. Siitonen A. (2006). Territorial waters of the Baltic Sea as a source of infections caused by *Vibrio cholerae* non-O1, non-O139: report of 3 hospitalized cases. *Diagn. Microbiol. Infect. Dis.* 54 1–6. 10.1016/j.diagmicrobio.2005.06.020 16368474

[B70] Luo Y. Ye J. Jin D. Ding G. Zhang Z. Mei L. (2013). Molecular analysis of non-O1/non-O139 *Vibrio cholerae* isolated from hospitalised patients in China. *BMC Microbiol.* 13:52 10.1186/1471-2180-13-52 PMC3605376 23497008

[B71] Mallard K. E. Desmarchelier P. M. (1995). Detection of heat-stable enterotoxin genes among Australian *Vibrio cholerae* O1 strains. *FEMS Microbiol. Lett.* 127 111–115. 10.1111/j.1574-6968.1995.tb07458.x 7737472

[B72] Martinez-Urtaza J. Huapaya B. Gavilan R. G. Blanco-Abad V. Ansede-Bermejo J. Cadarso-Suarez C. (2008). Emergence of asiatic *Vibrio* diseases in South America in phase with El Niño. *Epidemiology* 19 829–837. 10.1097/EDE.0b013e3181883d43 18854707

[B73] Matteucci G. Schippa S. Di Lallo G. Migliore L. Thaller M. C. (2015). Species diversity, spatial distribution, and virulence associated genes of culturable vibrios in a brackish coastal Mediterranean environment. *Ann. Microbiol.* 65 2311–2321. 10.1007/s13213-015-1073-6

[B74] Metzger L. C. Blokesch M. (2016). Regulation of competence-mediated horizontal gene transfer in the natural habitat of *Vibrio cholerae*. *Curr. Opin. Microbiol.* 30 1–7. 10.1016/j.mib.2015.10.007 26615332

[B75] Nelson E. J. Harris J. B. Morris J. G. Calderwood S. B. Camilli A. (2009). Cholera transmission: the host, pathogen and bacteriophage dynamic. *Nat. Rev. Microbiol.* 7 693–702. 10.1038/nrmicro2204 19756008 PMC3842031

[B76] Nigro O. D. Hou A. Vithanage G. Fujioka R. S. Steward G. F. (2011). Temporal and spatial variability in culturable pathogenic *Vibrio* spp. in lake Pontchartrain, Louisiana, following Hurricanes Katrina and Rita. *Appl. Environ. Microbiol.* 77 5384–5393. 10.1128/AEM.02509-10 21642406 PMC3147459

[B77] Nishiguchi M. K. (2000). Temperature affects species distribution in symbiotic populations of *Vibrio* spp. *Appl. Environ. Microbiol.* 66 3550–3555. 10.1128/AEM.66.8.3550-3555.2000 10919820 PMC92184

[B78] Nyholm S. Nishiguchi M. (2008). The evolutionary ecology of a sepiolid squid-*Vibrio* association: from cell to environment. *Vie Milieu Paris* 58 175–184. 20414482 PMC2857784

[B79] Oberbeckmann S. Fuchs B. M. Meiners M. Wichels A. Wiltshire K. H. Gerdts G. (2012). Seasonal dynamics and modeling of a *Vibrio* community in coastal waters of the North Sea. *Microb. Ecol.* 63 543–551. 10.1007/s00248-011-9990-9 22202887

[B80] Orata F. D. Keim P. S. Boucher Y. (2014). The 2010 cholera outbreak in Haiti: how science solved a controversy. *PLoS Pathog.* 10:e1003967 10.1371/journal.ppat.1003967 PMC3974815 24699938

[B81] Panicker G. Bej A. K. (2005). Real-time PCR detection of *Vibrio vulnificus* in oysters: comparison of oligonucleotide primers and probes targeting vvhA. *Appl. Environ. Microbiol.* 71 5702–5709. 10.1128/AEM.71.10.5702-5709.2005 16204478 PMC1265985

[B82] Panicker G. Myers M. L. Bej A. K. (2004). Rapid detection of *Vibrio vulnificus* in shellfish and Gulf of Mexico water by real-time PCR. *Appl. Environ. Microbiol.* 70 498–507. 10.1128/AEM.70.1.498-507.2004 14711681 PMC342803

[B83] Paz S. Bisharat N. Paz E. Kidar O. Cohen D. (2007). Climate change and the emergence of *Vibrio vulnificus* disease in Israel. *Environ. Res.* 103 390–396. 10.1016/j.envres.2006.07.002 16949069

[B84] Poloczanska E. Babcock R. Butler A. Hobday A. Hoegh-Guldberg O. Kunz T. (2007). Climate change and Australian marine life. *Oceanogr. Mar. Biol.* 45 407–478.

[B85] Ralph A. Currie B. J. (2007). *Vibrio vulnificus* and *V. parahaemolyticus* necrotising fasciitis in fishermen visiting an estuarine tropical northern Australian location. *J. Infect.* 54 e111–e114. 10.1016/j.jinf.2006.06.015 16890991

[B86] Ralston E. P. Kite-Powell H. Beet A. (2011). An estimate of the cost of acute health effects from food-and water-borne marine pathogens and toxins in the USA. *J. Water Health* 9 680–694. 10.2166/wh.2011.157 22048428 PMC5439350

[B87] Randa M. A. Polz M. F. Lim E. (2004). Effects of temperature and salinity on *Vibrio vulnificus* population dynamics as assessed by quantitative PCR. *Appl. Environ. Microbiol.* 70 5469–5476. 10.1128/AEM.70.9.5469-5476.2004 15345434 PMC520858

[B88] Rao A. Stockwell B. (1980). The Queensland cholera incident of 1977: 1. The index case^∗^. *Bull. World Health Organ.* 58 663–664. 6969138 PMC2395934

[B89] Rizvi A. V. Bej A. K. (2010). Multiplexed real-time PCR amplification of tlh, tdh and trh genes in *Vibrio parahaemolyticus* and its rapid detection in shellfish and Gulf of Mexico water. *Antonie Van Leeuwenhoek* 98 279–290. 10.1007/s10482-010-9436-2 20376562

[B90] Rogers R. Cuffe R. Cossins Y. Murphy D. Bourke A. (1980). The Queensland cholera incident of 1977: 2. The epidemiological investigation^∗^. *Bull. World Health Organ.* 58 665–669. 6969139 PMC2395928

[B91] Sawabe T. Ogura Y. Matsumura Y. Feng G. Amin A. K. M. R. Mino S. (2013). Updating the *Vibrio* clades defined by multilocus sequence phylogeny: proposal of eight new clades, and the description of *Vibrio tritonius* sp nov. *Front. Microbiol.* 4:414 10.3389/fmicb.2013.00414 PMC3873509 24409173

[B92] Seymour J. R. Doblin M. A. Jeffries T. C. Brown M. V. Newton K. Ralph P. J. (2012). Contrasting microbial assemblages in adjacent water masses associated with the East Australian Current. *Environ. Microbiol. Rep.* 4 548–555. 10.1111/j.1758-2229.2012.00362.x 23760900

[B93] Sterk A. Schets F. M. Husman R. Maria A. Nijs T. Schijven J. F. (2015). Effect of climate change on the concentration and associated risks of *Vibrio* Spp. in Dutch recreational waters. *Risk Anal.* 35 1717–1729. 10.1111/risa.12365 25809307

[B94] Strom M. S. Paranjpye R. N. (2000). Epidemiology and pathogenesis of *Vibrio vulnificus*. *Microb. Infect.* 2 177–188. 10.1016/S1286-4579(00)00270-7 10742690

[B95] Stypulkowska-Misiurewicz H. Pancer K. Roszkowiak A. (2006). Two unrelated cases of septicaemia due to *Vibrio cholerae* non-O1, non-O139 in Poland, July and August 2006. *Euro Surveill.* 11:E061130.2. 10.2807/esw.11.48.03088-en 17213560

[B96] Suh J.-Y. Birch G. Hughes K. (2004). Hydrochemistry in reclaimed lands of the 2000 Olympic games site, Sydney, Australia. *J. Coast. Res.* 20 709–721. 10.2112/1551-5036(2004)20[709:HIRLOT]2.0.CO;2

[B97] Sun C. Feng M. Matear R. J. Chamberlain M. A. Craig P. Ridgway K. R. (2012). Marine downscaling of a future climate scenario for Australian boundary currents. *J. Climate* 25 2947–2962. 10.1175/JCLI-D-11-00159.1

[B98] Takemura A. F. Chien D. M. Polz M. F. (2014). Associations and dynamics of Vibrionaceae in the environment, from the genus to the population level. *Front. Microbiol.* 5:38 10.3389/fmicb.2014.00038 PMC3920100 24575082

[B99] Tamura K. Stecher G. Peterson D. Filipski A. Kumar S. (2013). MEGA6: molecular evolutionary genetics analysis version 6.0. *Mol. Biol. Evol.* 30 2725–2729. 10.1093/molbev/mst197 24132122 PMC3840312

[B100] Thompson F. L. Iida T. Swings J. (2004). Biodiversity of vibrios. *Microbiol. Mol. Biol. Rev.* 68 403–431. 10.1128/MMBR.68.3.403-431.2004 15353563 PMC515257

[B101] Thompson J. R. Polz M. F. (2006). “Dynamics of *Vibrio* populations and their role in environmental nutrient cycling,” in *The Biology of Vibrios* eds Thompson F. L. Austin B. Swings J. (Washington, DC: ASM Press) 190–203.

[B102] Thompson J. R. Randa M. A. Marcelino L. A. Tomita-Mitchell A. Lim E. Polz M. F. (2004). Diversity and dynamics of a North Atlantic coastal *Vibrio* community. *Appl. Environ. Microbiol.* 70 4103–4110. 10.1128/AEM.70.7.4103-4110.2004 15240289 PMC444776

[B103] Thompson J. Roach A. Eaglesham G. Bartkow M. E. Edge K. Mueller J. F. (2011). Perfluorinated alkyl acids in water, sediment and wildlife from Sydney Harbour and surroundings. *Mar. Pollut. Bull.* 62 2869–2875. 10.1016/j.marpolbul.2011.09.002 21963084

[B104] Thornton B. Basu C. (2011). Real-time PCR (qPCR) primer design using free online software. *Biochem. Mol. Biol. Educ.* 39 145–154. 10.1002/bmb.20461 21445907

[B105] Tout J. Siboni N. Messer L. F. Garren M. Stocker R. Webster N. S. (2015). Increased seawater temperature increases the abundance and alters the structure of natural *Vibrio* populations associated with the coral *Pocillopora damicornis*. *Front. Microbiol.* 6:432 10.3389/fmicb.2015.00432 PMC4435422 26042096

[B106] Turner J. W. Good B. Cole D. Lipp E. K. (2009). Plankton composition and environmental factors contribute to *Vibrio* seasonality. *ISME J.* 3 1082–1092. 10.1038/ismej.2009.50 19421235

[B107] Vezzulli L. Brettar I. Pezzati E. Reid P. C. Colwell R. R. Höfle M. G. (2012). Long-term effects of ocean warming on the prokaryotic community: evidence from the vibrios. *ISME J.* 6 21–30. 10.1038/ismej.2011.89 21753799 PMC3246245

[B108] Vezzulli L. Pezzati E. Stauder M. Stagnaro L. Venier P. Pruzzo C. (2015). Aquatic ecology of the oyster pathogens *Vibrio splendidus* and *Vibrio aestuarianus*. *Environ. Microbiol.* 17 1065–1080. 10.1111/1462-2920.12484 24725454

[B109] Yong L. Guanpin Y. Hualei W. Jixiang C. Xianming S. Guiwei Z. (2006). Design of *Vibrio* 16S rRNA gene specific primers and their application in the analysis of seawater*Vibrio* community. *J. Ocean Univ. China* 5 157–164. 10.1007/BF02919216

[B110] Zorrilla I. Chabrillón M. Arijo S. Dìaz-Rosales P. Martìnez-Manzanares E. Balebona M. C. (2003). Bacteria recovered from diseased cultured gilthead sea bream (*Sparus aurata* L.) in southwestern Spain. *Aquaculture* 218 11–20. 10.1016/S0044-8486(02)00309-5

