# Supplementary material for: Spatiotemporal Dynamics of Vibrio spp. within the Sydney Harbour Estuary
Source: Front Microbiol. 2016 Apr 12;7:460. doi: 10.3389/fmicb.2016.00460 (PMC4829023; doi:10.3389/fmicb.2016.00460)
Supplement: FIGURE S1 — Distribution of the average seawater surface temperatures at Port-Hacking. [file Image_1.PDF]

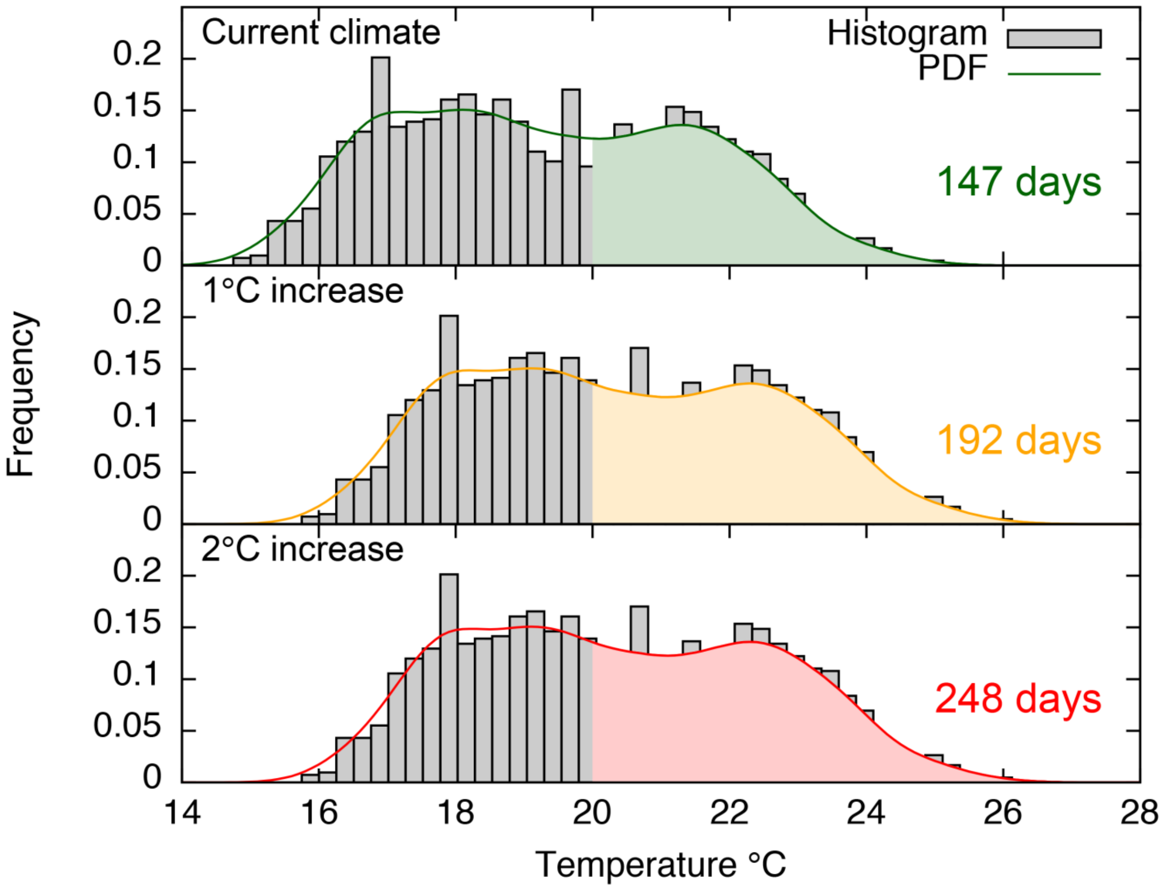

Figure S1: Distribution of the average seawater surface (0-10 m) temperatures (SST) at Port-Hacking (34°05'S 151°15'E) during 1953-2010 with prospect for warmer future. The highlighted area refers to the number of days in which average SST is above 20°C. (a) green - current climate measurements (b) orange - following sift of 1°C in the average SST (c) red - following sift of 1°C in the average SST. The 20°C threshold was chosen due to the fact that *V. cholerae*, *V. vulnificus* and *V. parahaemolyticus* all generally prefer warm water temperatures >20°C (Takemura et al., 2014).

Takemura, A.F., Chien, D.M., and Polz, M.F. (2014). Associations and dynamics of Vibrionaceae in the environment, from the genus to the population level. *Front. Microbiol*. **5**:38. doi: 10.3389/fmicb.2014.00038.
