# Supplementary material for: Spatiotemporal Dynamics of Vibrio spp. within the Sydney Harbour Estuary
Source: Front Microbiol. 2016 Apr 12;7:460. doi: 10.3389/fmicb.2016.00460 (PMC4829023; doi:10.3389/fmicb.2016.00460)
Supplement: TABLE S1 — Sample location and environmental associated data. [file Table_1.DOCX]

**Table S1: Sample location and environmental associated data.**

|  |  | **Average ± stdev µg L^-1^ (n=3)** | | | **Measurements taken in the field** | | | |
| --- | --- | --- | --- | --- | --- | --- | --- | --- |
| **Sample**  **location** | **Month** | **NH_4_^+^** | **NOx** | **NO_2_** | **Temp.**  **(°C)** | **DO**  **(mg L^-1)^** | **Salinity**  **(ppt)** | **Ph** |
| Chowder Bay | March | 27 ± 3.9 | 29.9 ± 0.7 | 12.4 ± 0.4 | 22.9 | 8.6 | 34.9 | 7.3 |
| Rozelle Bay | March | 24 ± 4.4 | 5 ± 13 | 14.1 ± 1.2 | 23.9 | 7.4 | 26.0 | 7.7 |
| Mort Bay | March | 30.6 ± 2.4 | 28.3 ± 0.9 | 12.6 ± 0.3 | 23.9 | 8.2 | 34.0 | 7.9 |
| Iron Cove | March | 33.3 ± 9.4 | 30.2 ± 4.1 | 12.4 ± 0.5 | 25.3 | 6.8 | 33.3 | 7.9 |
| Hen and Chicken Bay | March | 59.9 ± 7.5 | 26 ± 0.4 | 12.4 ± 0.4 | 25.8 | 7.4 | 32.0 | 7.8 |
| Rhodes | March | 116.4 ± 17.5 | 65.3 ± 16.3 | 14.1 ± 1.2 | 24.1 | 7.3 | 14.1 | 7.3 |
| Olympic Park | March | 313 ± 23.8 | 233.3 ± 32.9 | 22.6 ± 0.2 | 21.8 | 6.8 | 5.0 | 6.8 |
| Parramatta Park | March | 125.9 ± 13.1 | 197.6 ± 19.2 | 20.6 ± 1.4 | 24.0 | 8.2 | 0.0 | 7.0 |
| Chowder Bay | June | 68.7 ± 47.4 | 30.1 ± 1.5 | 13 ± 0.4 | 17.3 | 9.3 | 35.8 | 8.3 |
| Rozelle Bay | June | 59.2 ± 5.8 | 45.5 ± 2.2 | 13.6 ± 0.2 | 16.5 | 8.7 | 34.5 | 8.1 |
| Mort Bay | June | 30.2 ± 1.9 | 36.9 ± 0.9 | 13.4 ± 0.1 | 17.3 | 9.8 | 35.0 | 8.2 |
| Iron Cove | June | 118.7 ± 43.8 | 38 ± 1.8 | 13.2 ± 0.2 | 14.7 | 9.3 | 33.8 | 7.9 |
| Hen and Chicken Bay | June | 31.5 ± 2.7 | 32.7 ± 2.2 | 13 ± 0.4 | 15.2 | 10.0 | 34.6 | 8.1 |
| Rhodes | June | 510.1 ± 192.6 | 53.4 ± 5.7 | 0 ± 0 | 13.3 | 9.7 | 37.5 | 8.1 |
| Olympic Park | June | 485.3 ± 105.3 | 59.7 ± 4.3 | 0 ± 0 | 13.8 | 9.6 | 36.9 | 8.0 |
| Parramatta Park | June | 62.9 ± 3.3 | 31.8 ± 6 | 0 ± 0 | 13.6 | 7.0 | 27.5 | 7.4 |
| Chowder Bay | August | 125.3 ± 107.6 | 21.1 ± 29.1 | 48 ± 2.9 | 16.6 | 9.8 | 33.3 | 8.5 |
| Rozelle Bay | August | 71.7 ± 9.9 | 45.1 ± 8.9 | 7 ± 0.8 | 16.0 | 8.8 | 23.2 | 8.3 |
| Hen and Chicken Bay | August | 131.2 ± 54.7 | 83.1 ± 22.2 | 37.8 ± 44.7 | 13.9 | 10.2 | 7.8 | 8.0 |
| Rhodes | August | 161.9 ± 126.8 | 706.1 ± 905.6 | 15.7 ± 1.1 | 14.5 | 9.0 | 14.2 | 7.9 |
| Olympic Park | August | 173 ± 92.7 | 102.6 ± 38.3 | 18.3 ± 15.2 | 14.1 | 8.4 | 9.1 | 7.6 |
